# Sex differences in biomarkers of end-organ damage following exertional heat stroke in humans

**DOI:** 10.1152/japplphysiol.00463.2024

**Published:** 2024-09-19

**Authors:** Kari C. Goodwin, Gabrielle E. W. Giersch, Timothy A. Murray, David W. DeGroot, Nisha Charkoudian

**Affiliations:** ^1^Oak Ridge Institute of Science and Education, Oak Ridge, Tennessee, United States; ^2^United States Army Research Institute of Environmental Medicine, Natick, Massachusetts, United States; ^3^The Army Heat Center, Ft. Moore, Georgia, United States

**Keywords:** exercise, heat stress, military physiology, thermoregulation, women

## Abstract

Women are participating in military and athletic activities in the heat in increasing numbers, but potential sex differences in sequelae from exertional heat illness remain poorly understood. We tested the hypothesis that women suffering from exertional heat stroke (EHS) would have similar severity of organ damage biomarkers compared with men, as measured in a hospital setting. We studied women and men presenting with EHS to the emergency department at Fort Moore, GA. We measured creatinine (CR), creatine kinase (CK), alanine-transaminase (ALT), aspartate aminotransferase (AST), and estimated glomerular filtration rate (eGFR). Core temperature was also assessed by medical personnel. Biomarker data were obtained for 62 EHS cases (11 women). Men were significantly taller, and heavier, and had larger body mass index (BMI) and body surface area (*P* < 0.05 for all). The highest recorded body core temperature was not different between groups [women: 41.11°C (40.06, 41.67); men: 41.11°C (40.28, 41.72), *P* = 0.57]. Women had significantly lower peak CR [women: 1.39 (1.2, 1.48) m·dL^−1^; men: 1.75 (1.53, 2.16) mg·dL^−1^, *P* < 0.01] and peak CK [women: 584 (268, 2,412) U·L^−1^; men: 2,183 (724, 5,856) U·L^−1^, *P* = 0.02]. Peak ALT and AST were not different between groups; during recovery time points, ALT and AST were either similar or lower in women. Women spent approximately half as much time in the hospital following admittance compared with men. Our findings suggest that women may be less susceptible to organ injury resulting from EHS. Further research is necessary to understand the pathophysiology underlying these differences and how biomarkers of end-organ damage severity can differ between women and men following EHS.

**NEW & NOTEWORTHY** We studied otherwise healthy women and men after exertional heat stroke in a military training environment. Peak values for biomarkers of kidney and muscle damage were lower in women compared with men despite similar (highest recorded) body core temperatures. During recovery, organ damage markers were similar or lower in women. These sex differences may indicate differences in the pathophysiology of responses, but more work is needed to clarify specific mechanisms.

## Graphical abstract

**Figure:**
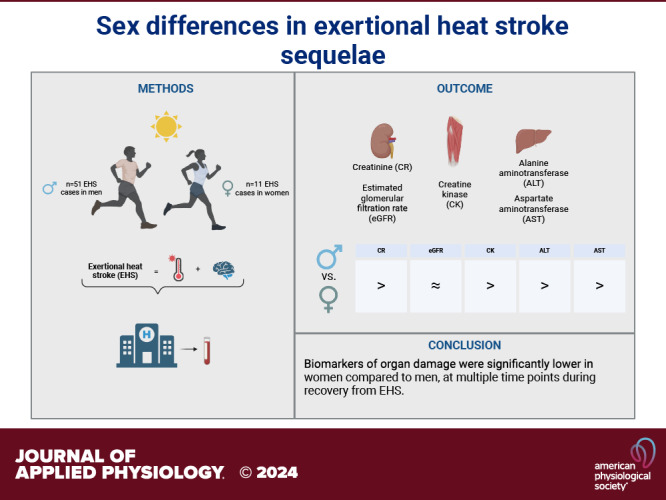

## INTRODUCTION

Exertional heat stroke (EHS), the most severe form of illness caused by exertion in the heat (1), is an ongoing threat to athletes, military personnel, and others who work or train in the heat. In 2023, 415 individuals across the U.S. Military suffered EHS, 54 of whom were women (2). For those engaged in outdoor, high-intensity activities, increasingly hot and humid summers resulting from climate change can increase risk. Due to a variety of intrinsic and extrinsic factors, EHS is not 100% preventable (1). In a military context, motivation plays a major role in the risk of EHS as training events (i.e., ruck marches and runs) are often timed or tied to the potential for promotion (3). Understanding physiological recovery from EHS in both sexes is vital, as women’s participation is increasing in military (4) and in athletic events where EHS risk is high. This need is also highlighted by the drastic underrepresentation of women in thermoregulatory research (5), such that our current understanding of the physiology and pathophysiology of such responses may be incomplete for ∼50% of the population.

Historically, women were often assumed to be at higher risk for EHS (6, 7), but recently, we showed that sex had no impact on the risk of developing EHS during military training (8). Although biological sex per se does not appear to influence the risk of EHS in a military cohort, physical factors, such as higher body mass index (BMI) and lower body surface area to mass ratio (BSA:mass^−1^), do increase EHS risk (8, 9). These body size variables are often, but not always, different between men and women. However, it is unclear if there are differences between men and women in post-EHS sequelae, including the potential for organ damage.

Whereas we have recently shown that sex is not an independent risk factor for EHS in humans, female sex hormones are known to have thermoregulatory impacts (10–13). Previously, investigators using a murine model observed enhanced resistance to EHS in female versus male mice (14) but greater myocardial metabolic dysfunction in female mice over male mice (15). In addition, female mice exhibited marked and prolonged epigenetic changes, including upregulation of heat shock proteins, not observed previously in male mice, which could indicate that female mice exhibit prolonged heat adaptations compared with male mice (16). Further research suggested a “protective” effect of the ovaries for EHS in female mice, suggesting sex hormones may impact EHS susceptibility (17). Although not directly translatable to humans, these recent findings highlight the importance of investigating potential differences in end-organ damage associated with EHS between men and women.

The organs most impacted by EHS are the kidneys, liver, and skeletal muscle (18). Damage is thought to be caused by ischemic strain within the splanchnic region on the liver and kidneys during long periods of high exertion when blood flow is predominantly shunted to the skin for heat dissipation and active skeletal muscle to support metabolic activity (19, 20). In a clinical setting, biomarkers of end-organ damage are commonly tested following EHS to assess the severity of injury to the kidneys (creatinine, CR; estimated glomerular filtration rate, eGFR), liver (alanine aminotransferase, ALT, and aspartate aminotransferase, AST) and skeletal muscle (creatine kinase, CK). These biomarkers are also valuable in assessing individuals’ ability to return to activity or return to duty (RTD) for military personnel. For example, at Ft. Moore, to be discharged from inpatient care, guidance is that three consecutive time points of biomarkers should be trending toward normal (21, 22). Currently, the criteria used to make decisions regarding RTD are based largely on data from men, highlighting the need for a better understanding of RTD considerations and recovery in women following EHS.

With this information as context, the purpose of the present study was to assess sex differences in biomarkers of end-organ damage between women and men diagnosed with EHS and transported to an emergency department at Ft. Moore, GA, between 2021 and 2023. A secondary aim was to evaluate the impact of these biomarkers on subsequent hospital admission and RTD. Based on existing data from humans, we hypothesized that there would be no difference in biomarkers associated with end-organ damage (CR, CK, ALT, AST, and eGFR) nor the timing of these biomarkers between young, otherwise healthy women and men following EHS.

## METHODS

### Ethical Approval and Participants

This study was approved by the U.S. Army Medical Research and Development Command Institutional Review Board (Protocol no. M-10872) and complied with all aspects of the *Declaration of Helsinki*, except for registration in a database. Volunteers provided written, informed consent as soon as they were medically stable if admitted to the hospital or at their first follow-up appointment at the Heat Clinic if they were discharged, usually the next business day. All subjects signed a Health Insurance Portability and Accountability Act (HIPAA) authorization and completed a demographic questionnaire.

Active-duty service members between the ages of 17 and 45 who were transported to the Emergency Department (ED) and diagnosed with EHS were eligible for inclusion. This study included 62 EHS cases: 10 women/51 men. Although *n* = 10 women were included in this analysis, there were *n* = 11 heat strokes within this sample. For one female volunteer, after her first EHS, she had resumed training and had a subsequent EHS 42 days later. For the purposes of the present analyses, it is being included as two separate EHS cases. Our study population consisting of 16% women was not statistically different from the active-duty Army population (∼16% active-duty women; Fisher’s exact test *P* > 0.05). Detailed participant biodemographic characteristics are found in Table 1.

**Table 1.** Subject demographics

	Women (*n* = 10)	Men (*n* = 51)
Age, yr	29 (21, 34)*	23 (21, 25)
Height, cm	162.6 (157.5, 172.7)*	177.8 (172.7, 182.9)
Weight, kg	65.83 (62.65, 76.73)*	83.99 (79.45, 93.98)
BMI, kg·m^−2^	25.24 (24.09, 26.52)*	27.37 (25.99, 28.98)
BSA, m^2^	1.70 (1.64, 1.92)*	2.02 (1.95, 2.14)
BSA:mass ratio, m^−2^·kg	0.0256 (0.0251, 0.0265)*	0.238 (0.228, 0.245)
Highest recorded body core temp, °C	41.11 (40.06, 41.67)	41.11 (40.28, 41.72)
Race *n* (%)		
Black	5 (50%)	7 (14%)
White	3 (30%)	31 (61%)
Other	2 (20%)	13 (25%)

Data presented are median and interquartile range (IQR), unless otherwise noted. *P < 0.05 vs. men. BMI, body mass index; BSA, body surface area; BSA:mass ratio, body surface area to mass ratio.

### Measurements and Experimental Design

Relevant laboratory data were retrieved from the electronic medical record. Blood samples were obtained for routine analysis upon admittance to the ED and in 6-h intervals if admitted to the hospital. Blood samples were also collected at follow-up appointments as deemed necessary by their primary care manager. All blood samples taken were part of a patient’s normal standard of care and were processed by hospital laboratory staff (Beckman Coulter DxC 700, Brea, CA, or VITROS 350 Chemistry Analyzer, Ortho Clinical Diagnostics, Raritan, NJ). All samples were collected in blood serum or plasma (depending on tube availability in the clinical settings) and centrifuged for 10 min at 3,500 rpm; serum was permitted to clot for >10 min prior to centrifugation. Serum creatinine concentration was used to calculate eGFR via the Chronic Kidney Disease Epidemiology Collaboration (CKD-EPI) equations for men and women (23). Baseline eGFR was assumed to be 75 mL·min^−1^·1.73^−2^ and eGFR following EHS was characterized using the Risk, Injury, Failure, Loss of kidney function, End-stage kidney disease (RIFLE) index, as previously described (24, 25).

Prehospital field care for all suspected heat casualties at Ft. Moore involves activating 911 for Emergency Medical System (EMS) transport to ED, obtaining a body core temperature, and initiating active cooling interventions (either ice sheets or cold-water immersion) as needed (26). Body core temperatures were obtained rectally by authorized medical personnel, either medics at the scene of collapse or EMS upon arrival to the field (Welch Allyn, Skaneateles Falls, NY; or Philips IntelliVue MP2, Suresnes, France; or LIFEPAK 15, Stryker, Redmond, WA). EMS documented all available core temperatures taken for the patient, the amount of time elapsed since the onset of EHS symptoms, the method of cooling, as well as length of time a patient was cooled. Highest recorded body core temperatures were utilized in this analysis with the recognition that the temperatures could have been significantly higher depending on duration and method of cooling prior to the arrival of medical personnel at the scene of collapse.

In the event, the unit medical staff was not on site to obtain a core temperature, cooling via ice sheets (ice water immersion is only allowed in the presence of continuous temperature monitoring by medics) was initiated immediately following the collapse or altered mental status (AMS) presentation, which would continue until EMS arrival (*n* = 10 women and *n* = 29 men underwent cooling prior to obtaining a core temperature). EMS obtained body core temperatures on every participant upon arrival. If core temperatures were still elevated over 38.89°C, continuous cooling by EMS occurred en route to the ED, utilizing a combination of ice sheeting (bed linens soaked in ice water), ice water lavage, and/or chilled saline. Serum sodium concentrations were also assessed in the field via i-STAT (Abbott Point of Care, East Windsor, NJ) to rule out hyponatremia, as AMS is also present in that condition. EHS diagnoses were confirmed by the Heat Clinic physician associate (26).

Upon the volunteer’s arrival to the ED, blood samples were collected based on the normal standard of care and were used to evaluate biomarkers of end-organ damage, including CR, eGFR, CK, ALT, and AST. The peak biomarker values for CR, CK, ALT, and AST, and minimum eGFR for each patient were used in this analysis, as well as timecourse data. Time bins were used to capture mean biomarker levels in 6-h intervals up to 32.99 h post injury, and then in 18-h intervals up to 104.9 h after injury. Mean biomarker values for each time bin were also used in this analysis. The time point of each biomarker’s peak was found in the electronic medical record and reported in hours postinjury, and the highest recorded body core temperature was obtained from EMS records. Peak time points for biomarkers, minimum eGFR, and highest recorded body core temperature were compared between sexes. The number of follow-up appointments and days from the point of injury to RTD is reported for each patient where that information was available and is reported in Table 2.

**Table 2.** Return to duty and follow-up visits

	Women	Men
# of follow-up visits	5 ± 2	4 ± 3
Days to RTD	74 ± 31	65 ± 24
Hospital admittance rate	82%	78%
Length of stay, days	1 (1)*	2 (1, 2)

Data presented are means ± SD, except length of stay, which is presented as median and interquartile range (IQR), *P < 0.05 vs. men. RTD, return to duty.

### Data and Statistical Analyses

Peak values for biomarkers, highest recorded body core temperature, and minimum eGFR values were included in this analysis, as well as time-binned data for the biomarkers. Time bins were utilized because biomarker measurements following discharge from the ED or inpatient care varied widely among volunteers, and this method allowed systematic batching of the data for analysis. In Figs. 2 and 3, each time bin represents the following range of hours post-EHS: bin 1 [0,3), bin 2 [3,9), bin 3 [9,15), bin 4 [15,21), bin 5 [21–27), bin 6 [27,33), bin 7 [33,51), bin 8 [51,69), bin 9 [69,87], and bin 10 [87,105). For this analysis, there was a significant portion of missing data in time bins 7–10. Therefore, for bins 7–10, laboratories were combined into 18-h intervals (for 33–104.99 h postinjury). As a result, there was not enough statistical power to assess those bins (denoted by dashed lines in Figs. 2 and 3); however, data are shown for their clinical relevance. Data were analyzed using Stata (SE Version 18.0, StataCorp LP, College Station, TX). Normality was assessed using a Shapiro–Wilk test. Where data were normally distributed, Student’s *t* tests were used to compare anthropometric variables and number of follow-up visits with a provider, and where not normally distributed, Mann–Whitney *U* tests were used primarily for peak biomarker measures, highest core temperature recorded, and length of hospitalization. Data not normally distributed are presented as median and interquartile range (IQR), where normally distributed variables are presented as means ± standard deviation (SD). Error bars in the figures represent the means ± standard error (SE). Linear mixed-effects regression models were used to evaluate the groups over the timecourse of each biomarker. For the linear mixed-effects models, we report the Akaike information criterion (AIC). Parameters are estimated using restricted maximum likelihood and the Kenward–Roger method was used to estimate the variance-covariance of the fixed effects (27). The linear mixed-effects model is shown in *Eq. 1*:

(1) $$y_{it}=\beta_{0}+\beta_{1}{\text{sex}}_{i}+\beta_{2}{\text{time}}_{t}+\beta_{2}{\text{sex}}_{i}\times {\text{time}}_{t}+\mu_{i}+\varepsilon_{it}$$

where $y_{it}$ represents CR, CK, ALT, and AST for subject i at time t. ${\text{sex}}_{i}$ is the sex of subject i, ${\text{time}}_{t}$ is a time-fixed effect to control for observed and unobserved variables that are constant across individuals but vary over time, and $\mu_{i}$ is a random effect for subject i  controlling for unobserved heterogeneity.

From the linear mixed-effects model, marginal contrasts were estimated for the outcome variable. Marginal contrasts show the difference in the predicted means of the outcome (${\hat{y}}_{it}$) at each time point between men and women. A chi-squared test was estimated on these marginal contrasts to assess if there were statistical differences between men and women at each time point. The regression results that were used to calculate the marginal contracts can be found in Table A1.

Multiple linear regression was conducted to evaluate the relative influence of sex, age, height, weight, body surface area (BSA), body surface area to mass ratio (BSA:mass^−1^), height, and weight on each biomarker. The linear regression model is shown in *Eq. 2*:

(2) $$y_{it}=\beta_{0}+\beta_{1}{\text{sex}}_{i}+\beta_{2}{\text{age}}_{i}+\beta_{3}{\text{height}}_{i}+\beta_{4}{\text{weight}}_{i}+\beta_{5}{\text{bsa}}_{i}+\beta_{6}\text{bsa}:{\text{mass}}_{i}+\phi_{t}+\varepsilon_{it}$$

where $y_{it}$ is the dependent variable for subject i at time t for CR, eGFR, CK, ALT, and AST. ${\text{sex}}_{i}$ is the sex of subject i, ${\text{age}}_{i}$ is the age of subject $i,{\;\text{height}}_{i}$ is the height of subject $i,{\;\text{weight}}_{i}$ is the weight of subject i, ${\text{bsa}}_{i}$ is the body surface area of subject i, ${\text{bsa:mass}}_{i}$ is the body surface area to mass ratio of subject i, and $\;\phi_{t\;}$ is a time fixed-effect that controls for time-invariant observable and unobservable differences.

Finally, it is important to understand the extent to which CR, CK, ALT, and AST differed from the upper limit of each biomarker’s clinical reference range, hereafter referred to as the “upper limit.” This is particularly important because men and women have different clinical reference ranges for the biomarkers utilized in this investigation. To evaluate the data observed relative to the upper limits, we created difference variables by subtracting the measured value from the upper limit for each sex (henceforth, this variable will be called “mean difference,” MD). An MD of zero means that individual *i* had a measurement that was identical to the upper limit of the reference range for that individual. This allowed us to assess how far each individual was from their respective upper limit at each time point. We evaluated the MD in two ways. First, we estimated the following linear mixed-effects model to test if the difference was statistically significant (different from zero). We estimated models restricted to men and women separately:

(3) $$y\_{\text{diff}}_{it}=\beta_{0}+\beta_{t}{\text{time}}_{t}+\varepsilon_{it}$$

where $y\_{\text{diff}}_{it}$ is the difference in the measured value of CR, CK, ALT, and AST at time *t* for subject *i* from the upper reference value. ${\text{time}}_{t}$ is a categorical variable for each time period. To assess the statistical significance of $y\_{\text{diff}}_{it}$, we estimated the predicted marginal effects from the model for each time period. Second, we tested whether there was a difference in the MD value between men and women. To do this, we estimated a linear fixed effects model with the same specification as *Eq. 1*, except the dependent variable was $y\_{\text{diff}}_{it}$. To test if there was a difference between men and women, we followed the same procedure outlined earlier. The results for these regressions can be found in Tables A2–A4.

For eGFR, proportions of the sample that fell into the different RIFLE categories were evaluated using Pearson’s chi-squared test. Categories included were “Normal” representing an increase or a decrease <25%, “Risk” representing a decrease between 25% and 49%, “Injury” representing a decrease between 50% and 74%, and “Failure” representing a decrease >75%. The number of follow-up visits and number of days until RTD were calculated from the electronic medical record. Statistical significance was set a priori at *P* < 0.05.

## RESULTS

### Participant Characteristics

In this sample, women were significantly older, shorter, and lighter, with lower BMI, BSA, and greater BSA:mass^−1^ as shown in Table 1. There were no differences in the highest recorded body core temperature or hospital admittance rates, but women had a shorter length of hospital admittance compared with their male counterparts (Table 2). Military ranks for women included 18% officers (*n* = 2), 18% noncommissioned officers (NCO, *n* = 2), and 64% enlisted (*n* = 7) versus men 37% officers (*n* = 19), 8% NCO, and 55% enlisted (*n* = 28).

### Peak Biomarker Results

**Figure 1.**
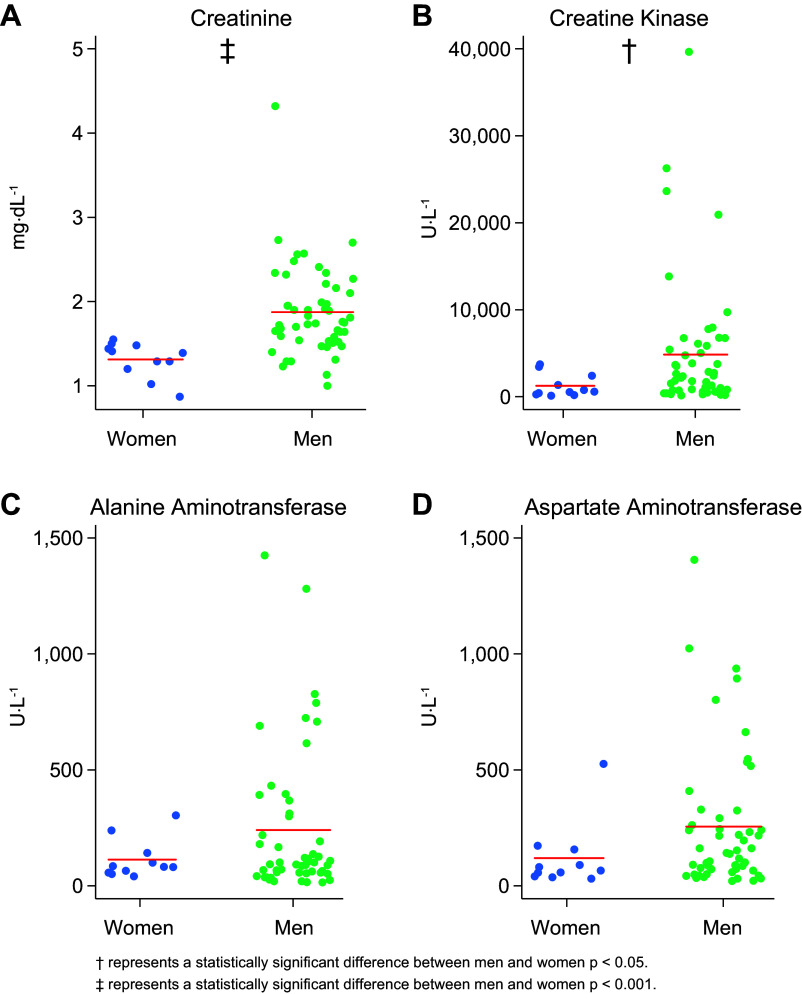
Peak biomarker measurements for men and women. These figures show the peak values for creatinine (*A*), creatine kinase (*B*), alanine aminotransferase (*C*), and aspartate aminotransferase (*D*) for each individual in the sample separated by sex along with the respective mean. *N* = 62 (51 men, 11 women). †Statistically significant difference between men and women where *P* < 0.05. ‡Statistically significant difference between men and women where *P* < 0.001.

Women had a significantly lower peak of CR [women: 1.39 (1.2, 1.48) mg·dL^−1^; men: 1.75 (1.53, 2.16) mg·dL^−1^, *P* < 0.01, Fig. 1*A*], relative to their male counterparts. There were no differences in the minimum value of eGFR between women and men [women: 54 (50, 63); men: 56 (42, 64), *P* = 0.58] or in stratification of eGFR RIFLE relative to the assumed baseline (Table 3). Peak CK was also lower in women [women: 584 (268, 2,412) U·L^−1^; men: 2,183 (724, 5,856) U·L^−1^, *P* = 0.02, Fig. 1*B*]. There were no differences in ALT values between groups [women: 82 (57, 142) U·L^−1^; men: 101 (57, 312) U·L^−1^, *P* = 0.48, Fig. 1*C*] and peak AST did not reach statistical significance [women 66 (41, 157) U·L^−1^; men: 142 (66, 292) U·L^−1^, *P* = 0.069, Fig. 1*D*]. There were no differences in timing of peak biomarker values for CR as all participants’ CR peaked upon admission to the ED as well as minimum eGFR (*P* > 0.99), and CK [women: 8.75 (0, 12.98); men: 12.82 (4.25, 20.73) h, *P* = 0.17]. The timing of the peak for ALT did not reach statistical significance [women: 54.85 (47.70, 72.18); men: 37.05 (22.03, 58.02) h, *P* = 0.06]. There were no differences in the timing of peak AST between sexes [women: 27.37 (0, 47.70); men: 25.42 (6.40, 37.87) h, *P* = 0.89].

**Table 3.** eGFR RIFLE criteria proportions

	Normal		Risk		Injury		Failure	
	Count	Percent	Count	Percent	Count	Percent	Count	Percent
Male	21	41.18	20	39.22	9	17.65	1	1.96
Failure	5	45.45	6	54.55	0	0.00	0	0.00
*P* value	0.794		0.350		0.132		0.640	

eGFR, estimated glomerular filtration rate; RIFLE, Risk, Injury, Failure, Loss of kidney function, End-stage kidney disease.

Multiple linear regression analysis was used to test for significant effects of sex, age, height, weight, BSA, and BSA:mass^−1^. For CR, ALT, and AST, there is a significant effect of sex when controlling for all other variables. For CR, age was also a significant predictor. For ALT, BSA:mass ratio showed a significant impact. Height, weight, BSA, and BSA:mass^−1^ were significant predictors for AST. The significant effect of sex disappears for CK, when controlling for other variables, but age, height, and weight were significant predictors. None of the variables influenced eGFR in the model. The regression results can be found in Table 4.

**Table 4.** Linear regression results

	(1)	(2)	(3)	(4)	(5)	(6)	(7)	(8)	(9)	(10)
	CR	CR	eGFR	eGFR	CK	CK	ALT	ALT	AST	AST
Sex	0.37 (0.03)***	0.31 (0.05)***	−3.86 (2.61)	−2.35 (3.70)	2,741.10 (378.51)***	707.19 (538.56)	93.85 (14.80)***	81.41 (25.98)**	103.57 (15.45)***	61.00 (21.08)**
Age		−0.01 (0.00)**		−0.17 (0.25)		−289.34 (52.35)***		1.02 (1.87)		−2.92 (1.55)
Height, cm		−0.14 (0.10)		0.82 (5.00)		−3,373.10 (1,598.96)*		−26.69 (37.66)		−207.26 (56.11)***
Weight, kg		−0.15 (0.08)		3.39 (3.86)		−3,107.54 (1,286.43)*		21.69 (35.24)		−148.27 (46.34)**
BSA		16.42 (10.28)		−243.59 (500.69)		36,0944.69 (16,5840.60)*		1,097.07 (4,021.76)		20,800.80 (5,862.12)***
BSA:mass		148.62 (168.89)		1,242.08 (8,838.48)		2.18e+06 (2.61e+06)		181,493.78 (69,107.26)**		347,807.84 (92,011.34)***
Observations	462	462	454	454	442	442	452	452	452	452
*R*-squared	0.390	0.448	0.382	0.446	0.073	0.142	0.235	0.283	0.163	0.247

Robust standard errors in parentheses. All models include a time fixed effect. *P < 0.05, **P < 0.01, ***P < 0.001. ALT, alanine aminotransferase; AST, aspartate aminotransferase; BSA, body surface area; BSA:mass, body surface area to mass ratio; CK, creatine kinase; CR, creatinine, eGFR, estimated glomerular filtration rate.

### Timecourse Biomarker Results

**Figure 2.**
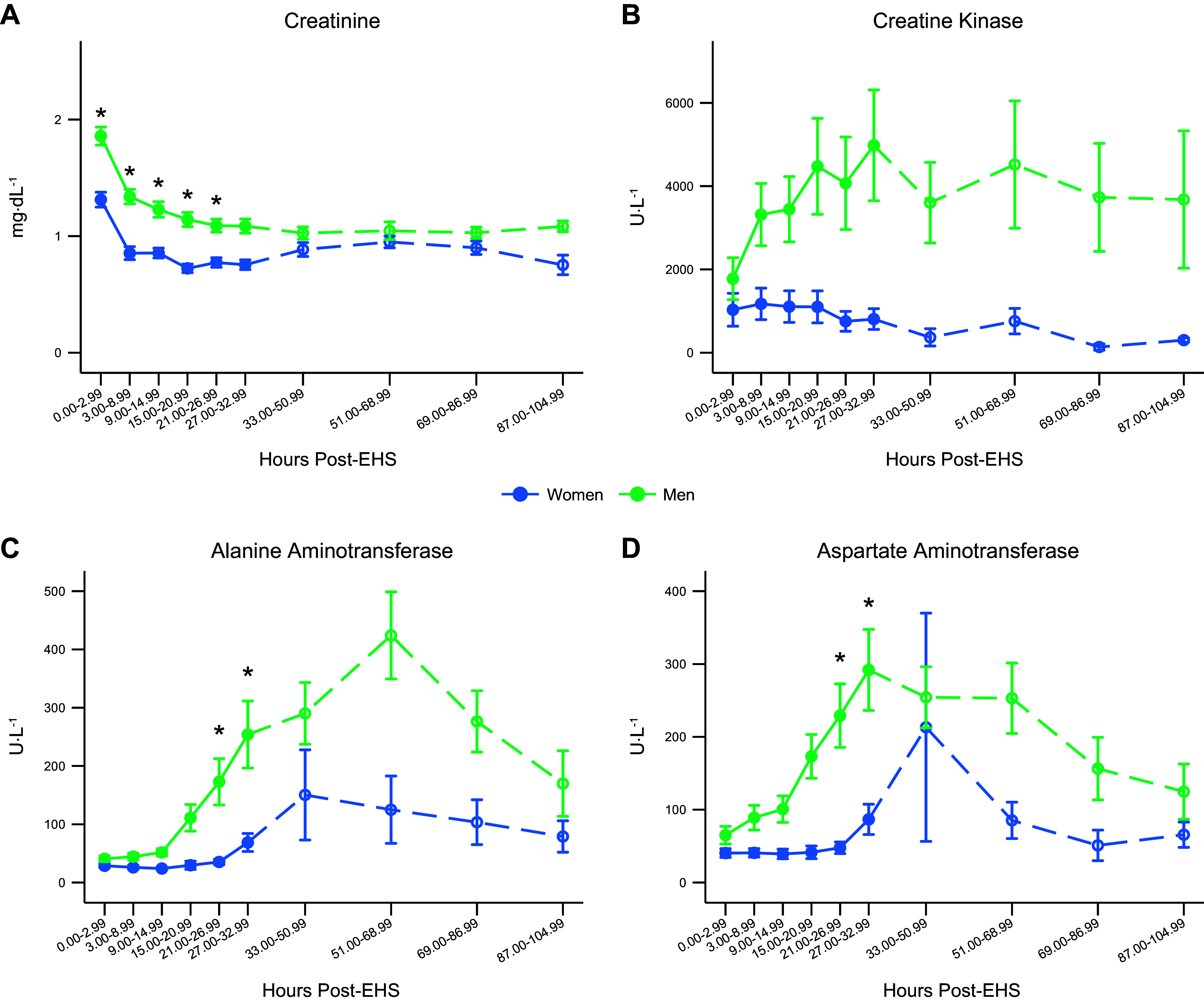
Mean values for creatinine (*A*), creatine kinase (*B*), alanine aminotransferase (*C*), and aspartate aminotransferase (*D*) over the timecourse for men and women. Error bars represent the standard error. For 33.00–104.99 h post-EHS, labs were combined into 18-h intervals due to a prevalence of missing data. As a result, there was not enough statistical power to assess those time points, but they are presented for clinical relevance and are denoted with a dashed line. *N* = 62 (51 men, 11 women). *Statistically significant difference between men and women *P* < 0.05. EHS, exertional heat stroke.

Women had significantly lower creatinine for time bins 1–5 compared with men (*P* < 0.05), Fig. 2*A*. There were no sex differences between any of the time points for creatine kinase (Fig. 2*B*). Women had significantly lower AST and ALT than men for time bins 5 and 6 (Fig. 2, *C* and *D*).

### Mean Difference from Upper Limit

**Figure 3.**
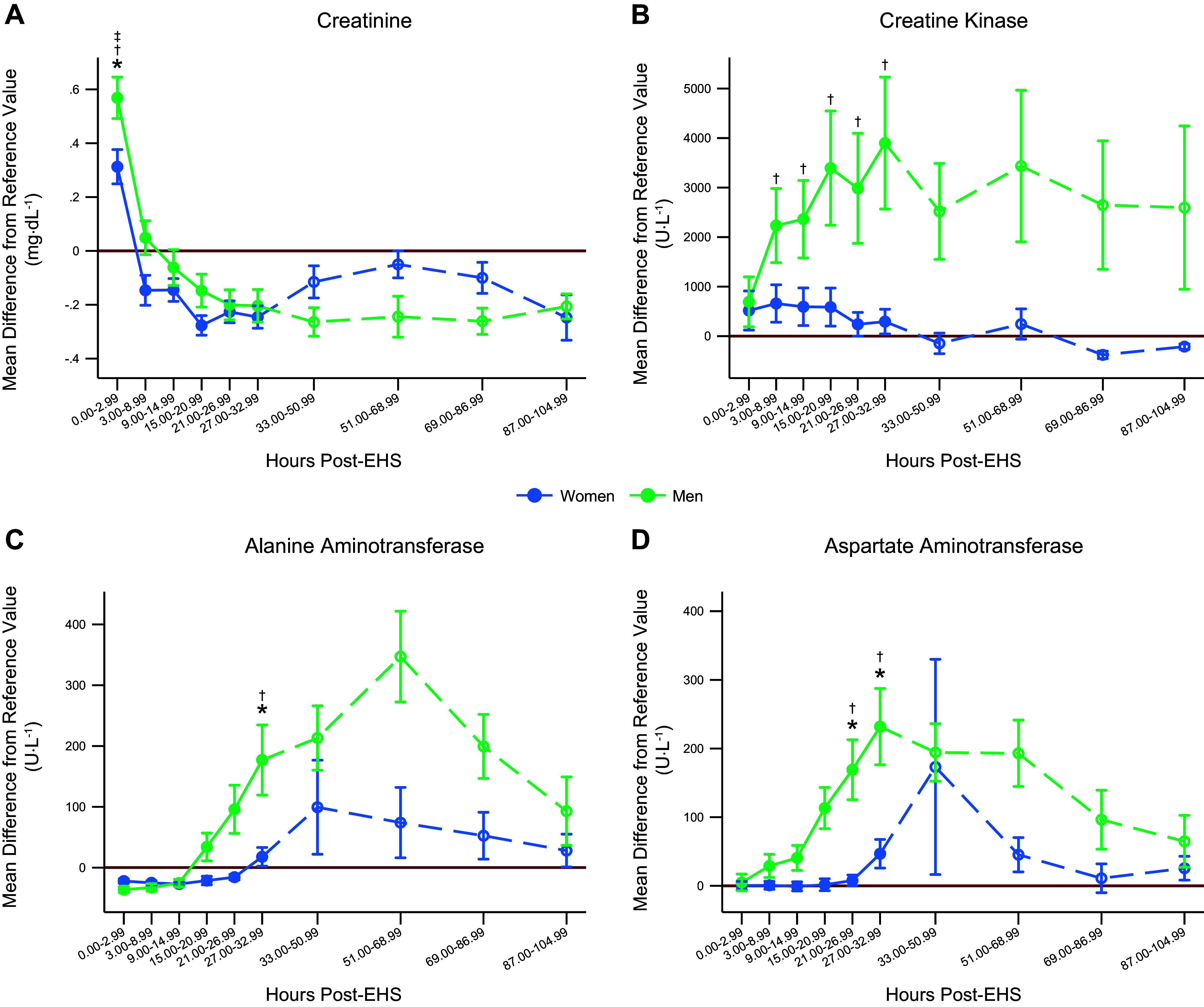
Mean difference (MD) from the upper limit of clinical reference value for men and women. *N* = 62 EHS, *n* = 51 men, *n* = 11 women analyzed using mixed-effects models. Areas of open circles and dashed lines area were not statistically analyzed due to missing data but are shown for clinical relevance. To calculate MD, the measured value for each individual was subtracted from the respective upper limit. An MD of zero means the measured value is equal to the upper limit. Since men and women have different limits, this allows us to assess how far the mean for men and women are from their respective upper limits and compare that difference between the sexes. *A*: MD data for creatine. *B*: MD data for creatine kinase. *C*: MD data for alanine aminotransferase. *D*: MD data for aspartate aminotransferase. For 33.00–104.99 h post-EHS, labs were combined into 18-h intervals due to a prevalence of missing data. As a result, there was not enough statistical power to assess those time points, but they are presented for clinical relevance and are denoted with a dashed line. *N* = 62 (51 men, 11 women). *Statistically significant difference between men and women *P* < 0.05. †Statistically significant difference from the upper limit for men *P* < 0.05. ‡Statistically significant difference from the upper limit for women *P* < 0.05. EHS, exertional heat stroke.

The mean difference (MD) for CR is found in Fig. 3*A*. Men had a greater MD compared with women. For both men and women, the MD for CR was significantly greater than zero at time bin 1. MD data for CK are found in Fig. 3*B*. Men had a significant MD (greater than zero) for time bins 2–6. The MD for CK was not different between men and women and, for women, was not statistically greater than zero. MD data for ALT are shown in Fig. 3*C*. Men had significantly higher MD compared with women at time bin 6. Women had MD values significantly greater than zero at time bins 4–6, and for men, this occurred at time bins 5 to 6. AST MD data are shown in Fig. 3*D*. Men had a significantly higher MD compared with women at time bins 5 to 6. Men had an MD statistically greater than zero at time bins 4–6, and for women, the MD was greater than zero at time bin 6.

### Body Temperature Results and Hospitalization

**Figure 4.**
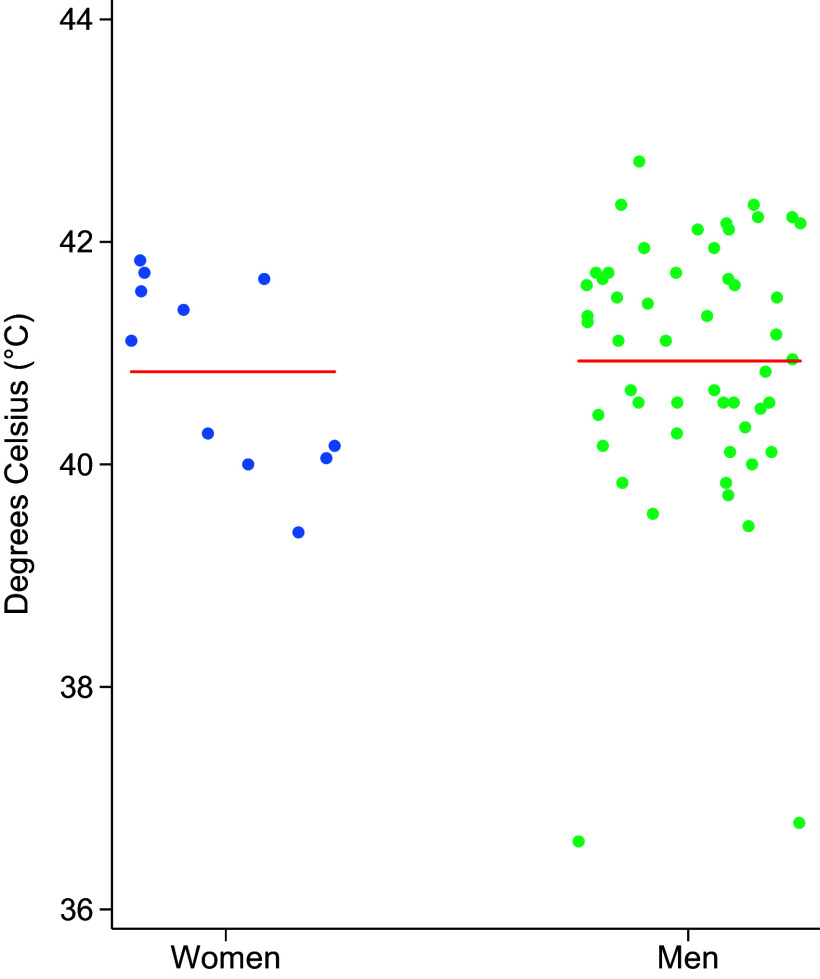
Maximum recorded core temperature for each individual, separated by sex, along with the respective mean for each group. *N* = 62 (51 men, 11 women). †Statistically significant difference between men and women where *P* < 0.05. ‡Statistically significant difference between men and women where *P* < 0.001.

The highest recorded body core temperature was not different between groups [women: 41.11°C (40.06, 41.67); men: 41.11°C (40.28, 41.72), *P* = 0.57, Fig. 4]. Body core temperatures were taken after the initiation of active cooling in 91% of the EHS case for women (*n* = 10), and in 57% of the cases for men (*n* = 29). Length of hospitalization was greater in men relative to women; there were no differences in the number of follow-up visits or RTD, as shown in Table 2.

## DISCUSSION

To our knowledge, this is the first study in humans to evaluate sex differences and timecourse of clinical biomarkers of organ damage in humans immediately following exertional heat stroke. Our major new findings were that peak values for biomarkers of kidney and muscle damage following EHS were significantly higher in men compared with women. Interestingly, there were no sex differences in the highest recorded body core temperature recorded for the EHS cases, suggesting similar EHS “severity” despite the differences observed in CR, CK, ALT, and AST (28, 29).

During recovery time points, values for CR, ALT, and AST were either similar or lower in women (see Fig. 2, *A, C*, and *D*). Length of hospitalization was greater in men relative to women, which may have been related to the higher CR and CK values observed in men. The results of the linear regression show a distinct effect of sex even when controlling for age, height, weight, BSA, and BSA:mass^−1^ for CR, ALT, and AST. The significant effect of CK did not appear to be related to sex, but rather related to body size. This provides an interesting context to these findings that sex is still a significant factor for CR, AST, and ALT, despite no significant differences in peak AST and ALT, even when controlling for body size.

Within each of these biomarkers, it is important to recognize the differences in clinical reference ranges between men and women. We evaluated the mean difference from the upper limit for men and women for CR, CK, ALT, and AST (i.e., Fig. 3). These findings provide valuable clinical context showing when men and women statistically exceeded their reference ranges, and when the mean difference differed between men and women. For CR, men had a significantly greater mean difference compared with women. Men had significantly higher absolute and relative CR increases following EHS compared with women. For CK specifically, these differences in the upper limit are likely due to increased muscle mass (30). Interestingly, only men statistically exceeded their upper limit and they did not have a significantly higher mean difference compared with women. Women also did not statistically exceed their upper limit for CK. Men had significantly higher absolute and relative ALT compared with women at ∼27 h post-EHS, with higher absolute and relative AST compared with women from 21 to 32.99 h following EHS. Utilizing recent guidance for eGFR as an assessment of kidney function, we observed no differences in minimum eGFR measured or the categorization of eGFR change, relative to an assumed baseline, between men and women.

We were surprised to note that the biomarker differences above occurred despite similar levels of (highest recorded) core temperatures between women and men. These results have several potential interpretations. First, it is possible that women may be less susceptible to acute organ damage following exertional heat stroke, similar to recent findings suggesting female mice showed higher resiliency against EHS (14, 17), as well as prolonged epigenetic changes and an upregulation in heat shock proteins, when compared with their male counterparts (16). An upregulation of heat shock proteins has been shown to be associated with greater heat acclimatization and could contribute to the decreased susceptibility of female mice to EHS (17), some of which may help to explain some of the differences in responses observed following EHS between the sexes in humans. Second, women tend to have lower BMI and muscle mass and higher BSA:mass ratios compared with men, factors that have been observed to influence EHS risk (8, 9). Third, women may have augmented organ blood flow due to differences in sympathetic neurovascular control mechanisms that might result in less vasoconstriction for a given sympathoexcitation during heat illness (31). However, we recognize the limitations of not controlling for the timing of this measurement, and the nature of the present observational study did not allow us to perform in-depth measurements to evaluate any of these possibilities.

Although no differences were observed in core temperatures in this investigation, due to the nature of the prehospital cooling protocols, the core temperatures observed in this study may be lower than the true peaks reached by individuals at the time of the collapse, especially for women who received cooling prior to EMS core temperatures in 91% of cases. Depending on the presence of on-site medics or EMS arrival, the timing of body core temperature recording varied on a case-by-case basis, and in 91% of women, body core temperatures were obtained after cooling interventions had taken place, suggesting that for 91% of women, the peak body core temperature was likely higher than we have on record, and the same was true for 57% of men.

Women spent approximately half as much time in the hospital following admittance; this may have been due in part to a less severe presentation of organ damage, but we are unable to directly conclude this based on the data from this study. The hospital standard of care is that patients with EHS are discharged following three subsequent down-trending biomarker values, so while all values do not have to be completely resolved (to “normal” levels) upon hospital discharge, they need to be generally trending in that direction. During the inpatient stay, blood samples are drawn approximately every 6 h and discharge protocols take CR, CK, ALT, and AST into account. It may be, therefore, that patients who initially had lower biomarker levels of end-organ damage would be discharged sooner. It is also possible that women were quicker to resolve biomarkers of end-organ damage compared with men, but we do not have the resolution with our present data to address this possibility. This highlights the need for more research investigating the severity of end-organ damage biomarkers and the mechanisms for returning to normal.

Given that women and men may have different ranges of what is considered clinically “normal” regarding these biomarkers, investigating the impact on treatment is warranted. In a military training environment, where diagnosis and treatment often dictate a return to duty timelines, sex differences in the severity of biomarkers of end-organ damage could impact lost duty time. Given that women have less severe biomarkers of end-organ damage compared with men, there could be a bias toward earlier hospital discharge in women. The clinical ranges considered “normal” need to be confirmed to ensure the safest approach to diagnosing, treating, and returning women soldiers to duty. It is possible that these laboratory values are telling a portion of a more complex story and that baseline levels of biomarkers are different between men and women and that these differences are not suggesting less severe organ damage in women. Further work is needed to understand how these differences in biomarkers impact recovery from EHS in women. Elevated CK and CR in men relative to women also highlight an important anthropometric factor, greater muscle mass in men compared with women, which likely contributes to differences in CK at baseline. However, greater muscle mass does not necessarily equate to greater damage following EHS, particularly given that our measures are relative to blood volume, where men, on average, have greater blood volumes than women as well.

Our data showed significantly longer hospitalization in men, but no difference between the number of follow-up appointments or the days until RTD. However, this should be considered carefully in subsequent care of EHS patients who are women, not to overlook the severity of their injury, relative to the biomarker levels following EHS. In addition, RTD in the Army is governed by Army Regulation 40-501 (AR-40-501, Table 3-2), which specifies 10 wk of duty restrictions and a gradual return to activity for optimal recovery and does not consider recovery of biomarkers associated with EHS, thus no differences between RTD in men and women is unsurprising in this cohort.

Future research would benefit from exploratory studies identifying alternative biomarkers that might more comprehensively reflect the extent of damage following exertional heat stroke and could help to better elucidate whether these findings are incidental or reflect a different pathophysiological response between the sexes. Additional biomarkers could be measures of kidney function sampled from urine collection, which is not part of the normal standard of care (and thus was not included in the present investigation).

## LIMITATIONS

Although the present results provide novel evidence of sex differences in the severity of EHS sequelae, our observational study design had some inherent limitations. The highest measured core temperature that we reported were not necessarily representative of peak temperature. Since we were following patients during normal standard of care, the measured values often occurred after the initiation of cooling, as this is the standard of care for field operations for suspected heat casualties. In addition, given the differences in hospitalization time between men and women, the number of serial samples in men is greater than in women. This is because, after discharge from inpatient care, blood samples are only collected at follow-up visits if deemed necessary by the treating physician or provider, not every 6 h as they are for inpatient care, this contributed to missing data and decreased resolution farther out from the time of injury. In addition, we were not able to measure certain variables that would have been useful in contextualizing these data (e.g., lean body mass) and clinical data surrounding volunteer treatment in the ED, or while inpatient. Since these volunteers entered the ED as heat casualties, additional measures beyond the normal standard of care were not possible in this investigation and would be beneficial for future research. In addition, since blood samples were only collected after the EHS event, there is no actual baseline eGFR for comparison and a baseline eGFR of 75 mL·min^−1^·1.73^−2^ was assumed. This assumption assumes “normal” kidney function in all volunteers.

For core temperature, out of the 62 EHS cases, two volunteers had normal core temperatures recorded with the presentation of AMS and EHS symptoms (*n* = 2 male, 36.61°C, and 36.78°C). These individuals had both received ice sheet cooling interventions in the field before EMS arrival (for 38 and 12 min, respectively) and temperature measurements. Sodium levels were assessed and determined to be normal to rule out hyponatremia. We were able to confirm with EMS and the Army Heat Center provider that these were in fact EHS cases, despite the normal core temperatures on record, due to the extensive nature of their cooling and the medical histories of each case.

The number of days prior to RTD was calculated from entries in the medical record by primary care managers, and in this sample, data were missing for 45% of women (*n* = 5) and 45% of men (*n* = 23). The number of days to RTD was assumed based on the expiration of profiles (the military term for limited duty due to injury). Therefore, our observation of no sex differences in RTD days includes a high proportion of missing data and would benefit from additional follow-up work.

### Conclusions

In the present cohort of young healthy individuals who were hospitalized with EHS, we found that biomarkers of organ damage were significantly lower in women compared with men, at multiple time points during recovery from EHS. Although the highest recorded temperatures were similar between groups, a greater proportion of premeasurement cooling in the women means that their actual peak core temperature was likely higher, making it even more striking that their markers of kidney and muscle damage were lower than the men. Further research is necessary to understand the pathophysiology underlying these differences and their implications for the understanding and treatment of exertional heat stroke in women and men.

## DATA AVAILABILITY

Data will be made available upon reasonable request.

## GRANTS

This work was supported by Army Medical Research and Development Grants MO210141 (to N. Charkoudian) and MO21041 (to D. W. DeGroot).

## DISCLAIMERS

The views, opinions, and/or findings contained in this article are those of the authors and should not be construed as an official United States Department of the Army position, or decision, unless so designated by other official documentation. Approved for public release, distribution unlimited. Citations of commercial organizations and trade names in this report do not constitute an official Department of the Army endorsement or approval of the products or services of these organizations.

## DISCLOSURES

No conflicts of interest, financial or otherwise, are declared by the authors.

## AUTHOR CONTRIBUTIONS

K.C.G., D.W.D., and N.C. conceived and designed research; K.C.G. and D.W.D. performed experiments; K.C.G., G.E.W.G., T.A.M., D.W.D., and N.C. analyzed data; K.C.G., G.E.W.G., T.A.M., D.W.D., and N.C. interpreted results of experiments; K.C.G., G.E.W.G., T.A.M., and N.C. prepared figures; K.C.G., G.E.W.G., D.W.D., and N.C. drafted manuscript; K.C.G., G.E.W.G., T.A.M., D.W.D., and N.C. edited and revised manuscript; K.C.G., G.E.W.G., T.A.M., D.W.D., and N.C. approved final version of manuscript.

## APPENDIX

The tables contained in this appendix contain the linear mixed-effect regression results that were used to generate the predicted marginal effects and contrasts. A chi-squared test was used if there were statistical differences between groups using the marginal contrasts.

Table A1 shows the results of the mixed-effect models that were used to test the difference between men and women shown in Fig. 2. Table A2 shows the results of the mixed-effect models that were used to test the differences between men and women in Fig. 3. Tables A3 and A4 show the results of the mixed-effect models that were used to test the differences between baseline and the mean difference for men and women, respectively.

**Table A1.** Mixed-effects regression results

	(1)	(2)	(3)	(4)
	CK	AST	ALT	CR
Men	745.94 (1,738.75)	24.52 (59.93)	11.90 (53.32)	0.55 (0.13)***
Time = 2	−21.59 (1,438.03)	−2.34 (62.85)	−2.85 (61.40)	−0.45 (0.10)***
Time = 3	−69.37 (1,438.07)	1.43 (62.85)	−1.22 (61.41)	−0.47 (0.10)***
Time = 4	−179.84 (1,502.40)	2.79 (69.26)	2.59 (67.64)	−0.53 (0.11)***
Time = 5	−422.50 (1,438.07)	9.93 (62.85)	10.03 (61.41)	−0.55 (0.10)***
Time = 6	−429.74 (1,500.77)	43.73 (65.61)	40.21 (64.11)	−0.55 (0.10)***
Men × Time = 2	1,124.37 (1,578.68)	29.37 (68.94)	13.21 (67.38)	−0.08 (0.11)
Men × Time = 3	1,964.00 (1,576.60)	35.68 (68.85)	10.74 (67.29)	−0.19 (0.11)
Men × Time = 4	2,624.07 (1,633.66)	102.30 (74.75)	67.57 (73.03)	−0.21 (0.12)
Men × Time = 5	2,547.39 (1,576.44)	149.01 (68.76)*	120.25 (67.21)	−0.25 (0.11)*
Men × Time = 6	2,926.75 (1,654.14)	168.32 (72.09)*	167.56 (70.45)*	−0.30 (0.11)**
Constant	1,030.18 (1,576.98)	40.45 (54.36)	28.82 (48.35)	1.31 (0.12)***
Observations	292	297	297	300
AIC	5,474	3,748	3,716	122

Mixed-effects models calculated using restricted maximum likelihood with the Kenward–Roger method. Standard errors in parentheses. AIC, Akaike information criterion; ALT, alanine aminotransferase; AST, aspartate aminotransferase; CK, creatine kinase; CR, creatinine. **P* < 0.05, ***P* < 0.01, ****P* < 0.001.

**Table A2.** Mixed-effects regression results used to test mean difference between men and women

	(1)	(2)	(3)	(4)
	CK	AST	ALT	CR
Men	175.94 (1,738.75)	4.52 (59.93)	−14.10 (53.32)	0.26 (0.13)*
Time = 2	−21.59 (1,438.03)	−2.34 (62.85)	−2.85 (61.40)	−0.45 (0.10)***
Time = 3	−69.37 (1,438.07)	1.43 (62.85)	−1.22 (61.41)	−0.47 (0.10)***
Time = 4	−179.84 (1,502.40)	2.79 (69.26)	2.59 (67.64)	−0.53 (0.11)***
Time = 5	−422.50 (1,438.07)	9.93 (62.85)	10.03 (61.41)	−0.55 (0.10)***
Time = 6	−429.74 (1,500.77)	43.73 (65.61)	40.21 (64.11)	−0.55 (0.10)***
Men × Time = 2	1,124.37 (1,578.68)	29.37 (68.94)	13.21 (67.38)	−0.08 (0.11)
Men × Time = 3	1,964.00 (1,576.60)	35.68 (68.85)	10.74 (67.29)	−0.19 (0.11)
Men × Time = 4	2,624.07 (1,633.66)	102.30 (74.75)	67.57 (73.03)	−0.21 (0.12)
Men × Time = 5	2,547.39 (1,576.44)	149.01 (68.76)*	120.25 (67.21)	−0.25 (0.11)*
Men × Time = 6	2,926.75 (1,654.14)	168.32 (72.09)*	167.56 (70.45)*	−0.30 (0.11)**
Constant	517.18 (1,576.98)	0.45 (54.36)	−22.18 (48.35)	0.31 (0.12)**
Observations	292	297	297	300
AIC	5,474	3,748	3,716	122

Mixed-effects models calculated using restricted maximum likelihood with the Kenward–Roger method. Standard errors in parentheses. AIC, Akaike information criterion; ALT, alanine aminotransferase; AST, aspartate aminotransferase; CK, creatine kinase; CR, creatinine. **P* < 0.05, ***P* < 0.01, ****P* < 0.001.

**Table A3.** Mixed-effects regression results to test mean difference for men

	(1)	(2)	(3)	(4)
	CK	AST	ALT	CR
Time = 2	1,101.95 (705.74)	27.02 (30.56)	10.39 (29.97)	−0.53 (0.05)***
Time = 3	1,894.56 (700.13)**	37.10 (30.33)	9.51 (29.75)	−0.66 (0.05)***
Time = 4	2,443.57 (695.10)***	105.06 (30.35)***	70.16 (29.76)*	−0.74 (0.05)***
Time = 5	2,124.38 (699.71)**	158.90 (30.09)***	130.26 (29.51)***	−0.80 (0.05)***
Time = 6	2,495.85 (753.63)***	211.97 (32.21)***	207.73 (31.58)***	−0.85 (0.05)***
Constant	693.12 (796.88)	4.97 (27.38)	−36.28 (24.34)	0.57 (0.06)***
Observations	243	249	249	252
AIC	4,656	3,217	3,191	120

Mixed-effects models calculated using restricted maximum likelihood with the Kenward–Roger method. Standard errors in parentheses. AIC, Akaike information criterion; ALT, alanine aminotransferase; AST, aspartate aminotransferase; CK, creatine kinase; CR, creatinine. **P* < 0.05, ***P* < 0.01, ****P* < 0.001.

**Table A4.** Mixed-effects regression results to test mean difference for women

	(1)	(2)	(3)	(4)
	CK	AST	ALT	CR
Time = 2	−36.38 (226.57)	−1.70 (11.25)	−2.77 (7.07)	−0.45 (0.04)***
Time = 3	−83.28 (226.58)	0.82 (11.25)	−0.85 (7.07)	−0.47 (0.04)***
Time = 4	−197.27 (236.69)	2.59 (12.38)	2.77 (7.79)	−0.53 (0.04)***
Time = 5	−436.41 (226.58)	9.32 (11.25)	10.40 (7.07)	−0.55 (0.04)***
Time = 6	−444.89 (236.41)	44.30 (11.74)***	40.32 (7.38)***	−0.55 (0.04)***
Constant	517.18 (291.84)	0.45 (8.40)	−22.18 (5.83)***	0.31 (0.04)***
Observations	49	48	48	48
AIC	705	423	387	−41

Mixed-effects models calculated using restricted maximum likelihood with the Kenward–Roger method. Standard errors in parentheses. AIC, Akaike information criterion; ALT, alanine aminotransferase; AST, aspartate aminotransferase; CK, creatine kinase; CR, creatinine. **P* < 0.05, ***P* < 0.01, ****P* < 0.001.
